# Analysis of Progression Toward Alzheimer’s Disease Based on Evolutionary Weighted Random Support Vector Machine Cluster

**DOI:** 10.3389/fnins.2018.00716

**Published:** 2018-10-08

**Authors:** Xia-an Bi, Qian Xu, Xianhao Luo, Qi Sun, Zhigang Wang

**Affiliations:** ^1^College of Information Science and Engineering, Hunan Normal University, Changsha, China; ^2^College of Mathematics and Statistics, Hunan Normal University, Changsha, China

**Keywords:** Alzheimer’s disease progression, functional connectivity, classification, disease-related brain regions, evolutionary weighted random support vector machine cluster

## Abstract

Alzheimer’s disease (AD) could be described into following four stages: healthy control (HC), early mild cognitive impairment (EMCI), late MCI (LMCI) and AD dementia. The discriminations between different stages of AD are considerably important issues for future pre-dementia treatment. However, it is still challenging to identify LMCI from EMCI because of the subtle changes in imaging which are not noticeable. In addition, there were relatively few studies to make inferences about the brain dynamic changes in the cognitive progression from EMCI to LMCI to AD. Inspired by the above problems, we proposed an advanced approach of evolutionary weighted random support vector machine cluster (EWRSVMC). Where the predictions of numerous weighted SVM classifiers are aggregated for improving the generalization performance. We validated our method in multiple binary classifications using Alzheimer’s Disease Neuroimaging Initiative dataset. As a result, the encouraging accuracy of 90% for EMCI/LMCI and 88.89% for LMCI/AD were achieved respectively, demonstrating the excellent discriminating ability. Furthermore, disease-related brain regions underlying the AD progression could be found out on the basis of the amount of discriminative information. The findings of this study provide considerable insight into the neurophysiological mechanisms in AD development.

## Introduction

Alzheimer’s disease (AD) is a devastating neuro-cognitive disorder of the human brain (Keren-Shaul et al., 2017; Kodis et al., 2018), which is characterized by the progressive loss of cognition and memory in elderly adults (Roy et al., 2016). Along with the aging of global population, the number of individuals suffering from AD will increase (Novak et al., 2017). It is predicted that there will be more than 100 million elderly people worldwide affected by AD by 2050 (Cortes-Canteli et al., 2015; Branca and Oddo, 2017). Therefore, the identification of AD and particularly its transitional phase, namely mild cognitive impairment (MCI), have received increasingly growing attentions in recent years (Cui et al., 2018). The individuals diagnosed with MCI could be further subdivided into the early MCI (EMCI) and late MCI (LMCI) (Lee et al., 2017) and the distinguishing criterions for EMCI and LMCI have been previously depicted in Alzheimer’s Disease Neuroimaging Initiative (ADNI) cohort (Nuttall et al., 2016). At present, there is still no therapy to prevent or reverse the AD pathological process (Forster et al., 2017). It is hence important to develop a new approach that could identify different stages of AD to enhance the understanding of AD pathophysiological progression, which is helpful to the preclinical AD studies.

A great deal of neuroimaging techniques could be utilized to image human brain function and structure, e.g., diffusion tensor imaging (DTI), magnetic resonance spectroscopy (MRS), electroencephalogram (EEG), functional magnetic resonance imaging (fMRI), and so on (Busato et al., 2016; Thanh Vu et al., 2017). Due to the advantages of high temporal and spatial resolutions, fMRI especially resting-state fMRI have gained increasingly growing popularities in the investigation of the whole-brain neural connectivity recently (Goense et al., 2016). As an advanced brain imaging technology, resting-state fMRI has shown a great potential in providing comprehensive information to achieve a high level of identification of the neurological diseases (Phillips, 2012; Rosa et al., 2015). Accordingly, the application of non-invasive resting-state fMRI is highly advantageous to unfold the complexity of brain connectivity network and examine the brain dynamic changes from EMCI to LMCI to AD.

Machine learning (ML) technologies were extensively used in automatic pattern recognition based on imaging data (dos Santos Siqueira et al., 2014; Moradi et al., 2015; Wang et al., 2018; Zeng et al., 2018). In existing literature, there has been a widespread interest to utilize ML methods to classify different stages of AD. Nozadi et al. (2018) employed a random forest (RF) algorithm based on the whole-brain approach to achieve the accuracies of 72.5 and 81.7% for 164 EMCI versus 189 LMCI and 189 LMCI versus 99 AD respectively. Goryawala et al. (2015) reported the accuracies of 73.6 and 90.1% for 114 EMCI versus 91 LMCI and 91 LMCI versus 55 AD using the linear discriminant analysis (LDA). Jie et al. (2018) utilized the multi-kernel SVM and displayed a high accuracy of 78.8% classifying 56 EMCI from 43 LMCI. It is noteworthy that the discrimination between EMCI and LMCI is more challenging in comparison to LMCI and AD.

In order to improve the classification performances especially of EMCI and LMCI, and enhance the understanding of neuropathology in the AD progression, a new method of evolutionary weighted random SVM cluster (EWRSVMC) was presented in this paper to diagnose different stages of AD. The EWRSVMC combined multiple weighted SVM classifiers to make the final decision, which was believed to be considerably stable and robust compared to other individual classifiers such as artificial neural network and decision tree. In addition, the EWRSVMC employed a method of evolution to guide feature selection to explore the optimal feature set for better classification performance. We performed the experiment 1 for EMCI/LMCI classification and the experiment 2 for LMCI/AD classification, yielding high accuracies of 90 and 88.89% respectively using this new framework. Furthermore, the disease-related brain regions were ranked according to the corresponding optimal features’ frequencies and the top-ranked brain regions could be found out. On the one hand, several high-frequency brain regions [e.g., superior temporal gyrus (STG.R), insula (INS.L) and middle temporal gyrus (MTG.L)] are presented in the two groups of experiments at the same time, which suggested that these brain regions play crucial roles in the progression of AD. On the other hand, some brain areas displayed high frequencies only in one group of experiment [e.g., superior frontal gyrus (SFGmed.L) and olfactory cortex (OLF.R) in the experiment 1, and parahippocampal gyrus (PHG.L) and posterior cingulate gyrus (PCG.L) in the experiment 2], which facilitated to understand differences in disease progression. These findings are in agreement with the claims of the previous studies on AD (Douaud et al., 2013; Xiang et al., 2013; Zhu et al., 2014) and provide a novel perspective to AD progression’s neurophysiological mechanisms.

## Materials and Methods

### Subjects

The neuroimaging data we utilized in this study came from the ADNI cohort^1^ (Morris et al., 2014). We collected the resting-state fMRI data of 105 participants, which contained 42 EMCI patients (18 male, average age 72.34 years), 38 LMCI patients (23 male, average age 72.99 years) and 25 AD subjects (12 male, average age 74.59 years). Every participant had clinical dementia rating (CDR) scores and mini-mental state examination (MMSE) scores to ensure that the data was homologous. Chi-squared test was utilized for gender comparisons and two-sample *t*-test was utilized for age, MMSE and CDR comparisons. The detailed demographic information for the patient cohorts was listed in **Table 1**.

**Table 1:** Demographic information.

Variable (Mean ± SD)	EMCI	LMCI	AD	*P*-value
Male/Female	18/24	23/15	12/13	0.11^a^/0.33^b^
Age	72.34 ± 6.87	72.99 ± 7.79	74.59 ± 7.03	0.69^a^/0.41^b^
MMSE	28.10 ± 1.57	27.11 ± 2.44	21.24 ± 3.44	0.03^a^/0.00^b^
CDR	0.45 ± 0.22	0.54 ± 0.14	0.92 ± 0.31	0.04^a^/0.00^b^

*^a^The P-value of the comparison between the EMCI and LMCI. ^b^The P-value of the comparison between the LMCI and AD.*

All participants were asked to lie still in a Siemens TRIO 3 Tesla machine using the same scanning parameters as follows: 64 × 64 acquisition matrix; flip angle = 80°; echo time (TE) / repetition time (TR) = 30/3000 ms; pixel spacing Y/pixel spacing X = 3.3/3.3mm; 140 image volumes; 48 axial slices; 3.313 mm slice thickness with no gap. During the scan, all participants should close eyes but keep awake with thinking of nothings (Liu et al., 2018).

### Data Preprocessing

The same image preprocessing for EMCI, LMCI and AD patients was performed by utilizing the Data Processing Assistant for Resting State fMRI (DPARSF) toolbox (Dan et al., 2017). Briefly, the data was preprocessed in nine steps: converting the data into NIFTI format; exclusion of the first 10 volumes; slice-timing correction; realignment for head movement compensation; normalization; smoothing (utilizing a Gaussian kernel); removing linear trend; temporal band-pass filtering; 9) regressing out the nuisance signals.

### Functional Connectivity Features

The brain is a dynamic system constructed by large-scale complex networks comprised of the connections between different brain regions (Braun et al., 2015). In this paper, we employ a popular automated anatomical labeling template (Rolls et al., 2015) to divide the cerebrum into 90 brain areas (45 for left and right hemisphere respectively). A representative resting-state fMRI signal for each brain region is generated by averaging the time series of voxels within each of 90 brain regions. The Pearson correlation coefficient between the representative signals of each pair of the brain regions is computed and treated as a proxy of functional connectivity (FC) (Noble et al., 2017). As a result, a total of 4005 (80 × 90/2) FCs are obtained for each subject and served as predictor features for the proposed EWRSVMC algorithm, which is considered to be a promising approach.

### The Evolutionary Weighted Random SVM Cluster

#### EWRSVMC Design

Machine learning techniques are widely used for pattern recognition (Zeng et al., 2017), among which the SVM model has received increasing popularities in the analysis of neurological disease based on the high-dimensional imaging data recently. Nevertheless, utilizing the single SVM classifier is too challenging to achieve excellent diagnostic performance due to the noise of brain imaging data. Bi et al. (2018) put forward a random SVM cluster (RSVMC) in which multiple SVM classifiers are combined for a final decision-making, which outperforms an individual SVM classifier. But, it could not be ignored that the diagnostic power of each individual classifier in the ensemble classifier may be greatly differential from others. The previous method of RSVMC ignores the fact that the individual SVM classifier with relatively high training error is likely to perform wrong voting on the new samples, which is likely to degrade the discriminative ability. Accordingly, there still remains room for the improvement with respect to the RSVMC method.

**FIGURE 1:**
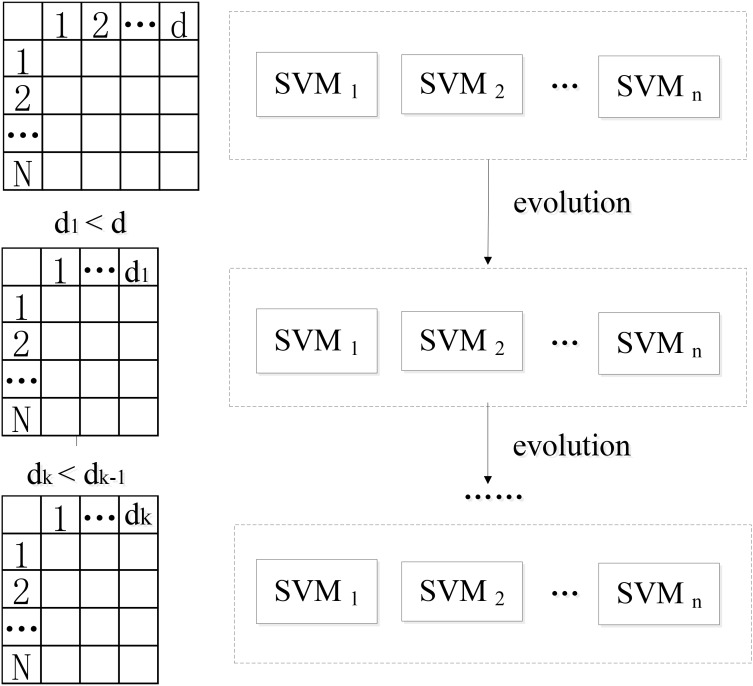
The idea of our proposed EWRSVMC.

This paper presents a novel algorithm of EWRSVMC with two successive steps, i.e., the construction and evolution of weighted ensemble of SVMs respectively. First, in order to reduce the influence of the weak classifiers on the voting, the classification accuracy of each SVM classifier is calculated using the validation set, which is regarded as a proxy of weight of every SVM classifier. The output of EWRSVMC is a weighted average of the outputs of multiple SVMs, which could further reduce classification error rate. Second, in order to select out the most discriminative features from a large-scale feature vector, the method of evolution is introduced to dynamically eliminate the redundant features for further improving final classification performance. The idea of our proposed architecture is showed in **Figure 1**, where each row and column corresponds to a subject and feature respectively in the left data matrixes.

We suppose *X* = {*x*_1_,...*x_k_*,...*x_n_*} ∈ *R^N × d^* as the connectivity features vectors where *N* and *d* are the numbers of all subjects and features. *y_i_* ∈ {+1, −1} is the response class label representing two different states (e.g., EMCL or LMCI). The construction of the weighted random SVM cluster is performed using the following steps:

(1) Step1: The available dataset *X* is divided into two data subset, i.e., a “training and validation” set and a test set respectively.
(2) Step2: Then, the training subset and feature subset are respectively obtained by randomly selecting partial samples from above “training and validation” set and partial features from total features to build an individual SVM model.
(3) Step3: The remaining validation subset is utilized for the estimation of diagnostic accuracy *W_l_* of *l*-th SVM, which is considered as a proxy of weight of the SVM.
  (1) $$W_{l}=\frac{T_{l}^{\mathrm{correct}}}{T_{L}}$$
  where $T_{l}^{\mathrm{correct}}$ denotes the number of validation samples correctly classified by *l*-th SVM classifier, *T_L_* represents the number of validation samples.
(4) Step4: The step 2 to step 4 are repeated for *n* times to build a weighted ensemble of *n* SVM classifiers.

Following the above steps, a weighted ensemble of multiple SVM classifiers could be constructed and then an approach of evolution is applied to the ensemble classifier to guide feature selection.

Specifically, the SVM classifiers whose classification accuracies are lower than 0.5 are first picked out from the weighted random SVM cluster and considered as weak classifiers. Similarly, the remaining SVM classifiers are regarded as strong classifiers due to the good performance. Then the features selected by these weak classifiers are found out and the weights corresponding to the common features are accumulated. The total weight of each feature in weak classifiers is denoted as *Tw_j_*:

(2) $${\mathrm{Tw}}_{j}=\sum_{l=1}^{p}w_{l,j}$$

where *p* is the number of weak classifiers; *w_l,j_* represents the weight of the *j*-th feature corresponding to *l*-th weak classifier.

**FIGURE 2:**
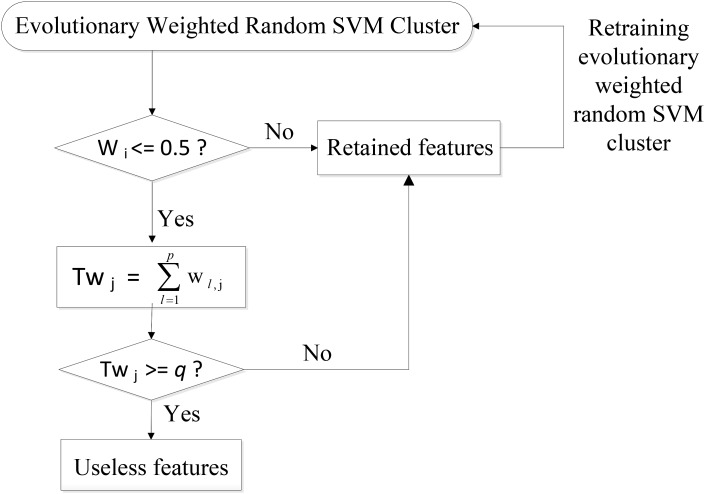
Feature selection procedure of the EWRSVMC.

Next, we remove the features whose total weight *Tw_j_* exceeds a certain threshold *q*, because these features play crucial roles in the weak classifiers and are likely to make few contributions to the excellent performance of the overall system. As a result, we obtain the remaining features with lower total weights in the weak classifiers and all the features determined by the strong classifiers as an evolutionary feature set, leading to the reduced dimensionality of total feature space. Finally, the above-obtained evolutionary feature set is employed to rebuild a weighted random SVM cluster for the further reduction of feature dimensionality. This procedure is repeated iteratively until it reaches the times of evolutions we set. The optimal EWRSVMC with the highest accuracy during the evolution process could be found out and the features determined by this optimal EWRSVMC are considered as the optimal feature set. The feature selection procedure of the EWRSVMC is exhibited in **Figure 2**.

#### The Evaluation of the EWRSVMC

The EWRSVMC perform a weighted average of the outputs of multiple SVM classifiers, which could predict the class label of each new testing sample. To be specific, a new sample is firstly input into a EWRSVMC system and each individual SVM classifier performs a weighted vote in accordance with its accuracy dealing with the validation samples. Then the weighted voting values belonging to the same predicted label are added up. Lastly, the label having the highest voting value represents new sample’s final predicted label.

In this paper, we employ the three metrics, i.e., accuracy, sensitivity and specificity to estimate our proposed EWRSVMC’s final performances. The diagnostic accuracy *A_c_* stands for a fraction of correctly identified samples (Schröder et al., 2015):

(3) $$A_{c}=\frac{\mathrm{TP}+\mathrm{TN}}{\mathrm{TP}+\mathrm{FP}+\mathrm{FN}+\mathrm{TN}}$$

where *TP*, *FP, FN*, and *TN* respectively represents the number of true positives, false positives, false negatives and true negatives.

Sensitivity (*S_n_*) stands for a proportion of actual positive samples which are correctly identified (Mondal and Pai, 2014):

(4) $$S_{n}=\frac{\mathrm{TP}}{\mathrm{TP}+\mathrm{FN}}$$

Specificity (*S_p_*) stands for a proportion of actual negative samples which are correctly identified (Kumar and Helenprabha, 2017):

(5) $$S_{p}=\frac{\mathrm{TN}}{\mathrm{TN}+\mathrm{FP}}$$

#### The Application of the EWRSVMC

In the current study, we conducted multiple binary classifications, including EMCI vs. LMCI and LMCI vs. AD to confirm the performance of our proposed EWRSVMC using 4005 FCs as the raw features. In addition to optimizing the classification accuracy as with most existing studies, we also paid great attentions to exploring and analyzing the alterations of the brain in patients with different cognitive stages of AD. Accordingly, another sub-procedure for the exploration of the disease-related brain regions using the optimal features set was carried out. First, we detected the brain regions which are relevant to the optimal features in the EWRSVMC with the highest classification accuracy. Then, disease-related brain regions were sorted in a descending mode, which is consistent with their occurrence frequencies. The higher the frequencies are, the greater the abnormal degrees of the brain regions are.

#### Experiment Design

In this paper, we conducted the experiment 1 for EMCI vs. LMCI classification and the experiment 2 for LMCI vs. AD classification. Each group of experiment could be mainly divided into four parts:

(1) Division of data sets. A 3:1 ratio is set to divide entire resting-state data set into the “training and validation” set for training the EWRSVMC and the test set for examining the generalization ability of the overall system. Furthermore, a 2:1 ratio is set to subdivide the “training and validation” set into the training set for training the SVM classifier and the validation set for obtaining the weight corresponding to the SVM classifier.

(2) Building an ERWSVMC. Firstly, we randomly select $\sqrt{4005}$ ≈ 62 features from all 4005 features based on the training set to build a radial basis function (RBF) kernel SVM classifier. The kernel bandwidth σ and penalty parameter *C* for each SVM model are primarily set as 3 and *Inf* respectively. The number of initial base classifiers is set to 500 to get the weighted ensemble of SVMs. Then, we make the ensemble classifier evolves for 50 times. In each evolution, we find out the features selected by the weak classifiers and remove the features whose total weight *Tw_j_* exceeding the certain threshold *q = 7.* As a result, the EWRSVMCs with different evolution times are obtained.

(3) Finding out the optimal subset of features. We compute the diagnostic accuracies of the EWRSVMCs with different evolution times. The features selected by the optimal EWRSVMC having the lowest diagnostic error rate form the optimal features subset.

(4) Exploring the abnormal brain regions. We seek out the features with high discriminative ability in the optimal EWRSVMC, and then investigate the corresponding disease-related brain regions associated with these features.

## Results

### The Experiment 1

**FIGURE 3:**
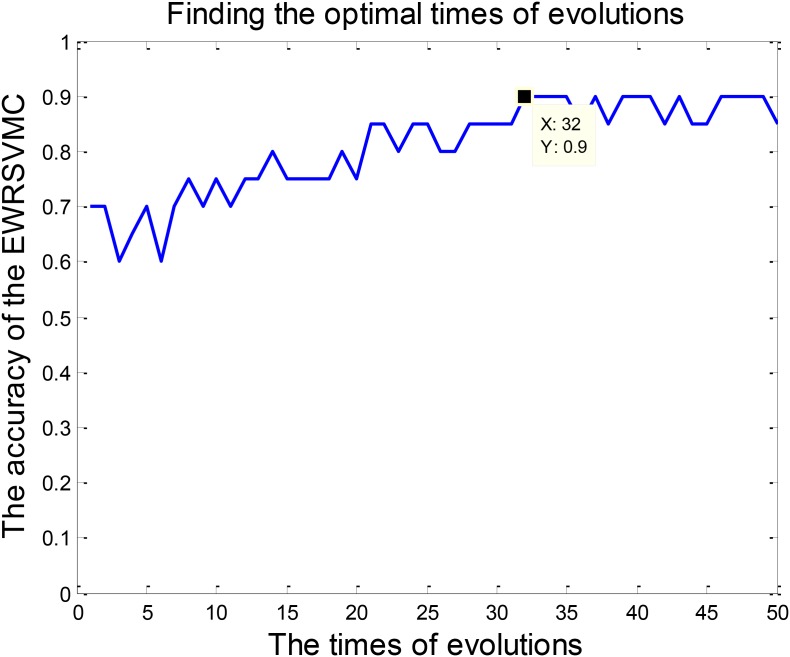
Finding the optimal times of evolutions in the experiment 1.

We investigated the performance of classification between EMCI and LMCI in the experiment 1. According to Section “Experiment Design,” we conducted 50 evolutions for the EWRSVMC. Consequently, the EWRSVMC yielded a maximum accuracy of 90% in the 32nd evolution (as shown in **Figure 3**), which suggested that 32 was the optimal times of evolutions. Meanwhile, a sensitivity of 90.9% and a specificity of 88.89% were achieved based on the optimal feature set. The experiment results showed that the novel framework could significantly enhance diagnostic performance for EMCI/LMCI classification in compared with some other existing algorithms.

**FIGURE 4:**
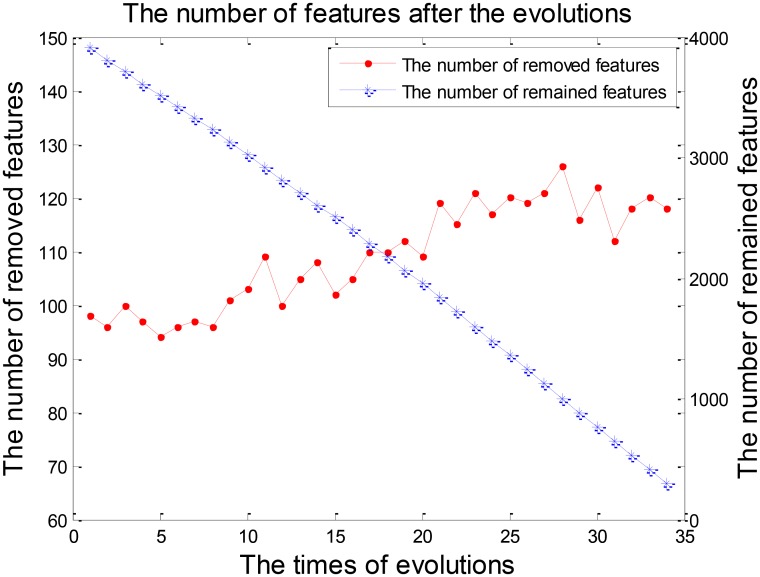
The number of features after each evolution in the experiment 1.

Feature selection was a crucial stage in our EWRSVMC algorithm classifying LMCI from EMCI and the process was shown in **Figure 4**. On the one hand, the number of removed features increased rapidly and exceeded 100 after two evolutions. Then it became gradually stable and fluctuated around 120. On the other hand, the number of remained features showed a trend of linear decline. There were 248 features left after completing the 32nd evolution, which constituted the optimal feature set and were utilized for subsequent study on the exploration of disease-related brain regions.

**FIGURE 5:**
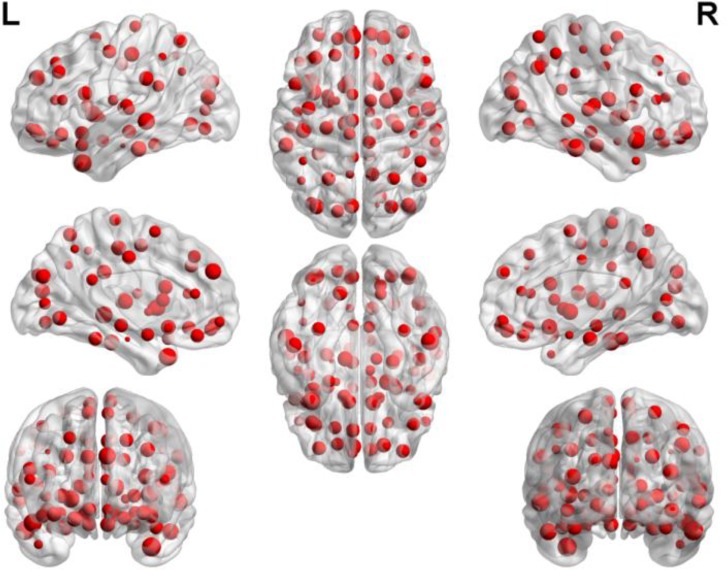
The locations of abnormal brain regions in the experiment 1.

By counting the high-frequency FCs, we could detect the most discriminative brain regions which were ranked in the **Table 2**. The brain regions exceeding the frequency of 10 comprise inferior temporal gyrus (ITG.R), temporal pole: middle temporal gyrus (TPOmid.L), temporal pole: superior temporal gyrus (TPOsup.R), middle temporal gyrus (MTG.L) and insula (INS.L). As seen from **Table 2**, some sub-regions of the temporal lobe showed higher frequencies compared to other regions, indicating the temporal lobe made an essential contribution to the evolution from EMCI to LMCI. The locations of brain regions were mapped in **Figure 5** and the size of the red node represented the degree of abnormality of the corresponding brain regions.

**Table 2:** The frequencies of the most discriminative brain regions in the experiment 1.

Frequency	Brain region
15	ITG.R
14	TPOmid.L
12	TPOsup.R MTG.L
11	INS.L
10	SFGmed.L PAL.R
9	OLF.R ITG.L

### The Experiment 2

**FIGURE 6:**
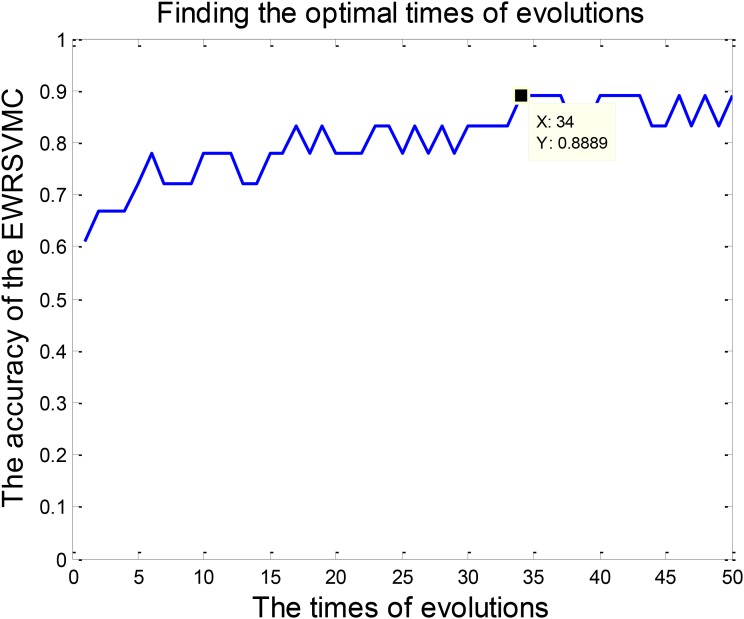
Finding the optimal times of evolutions in the experiment 2.

The classification of patients with LMCI and AD was carried out in the experiment 2. Similarly, 50 evolutions were performed and the EWRSVMC reported the highest accuracy of 88.89% in the 34nd evolution (please see **Figure 6**), which indicated that 34 was the optimal times of evolutions in LMCI/AD classification. At the same time, the optimal EWRSVMC achieved 85.71% sensitivity and 90.9% specificity. The encouraging performances demonstrated the potential of our new framework for the diagnosis of AD dementia.

**FIGURE 7:**
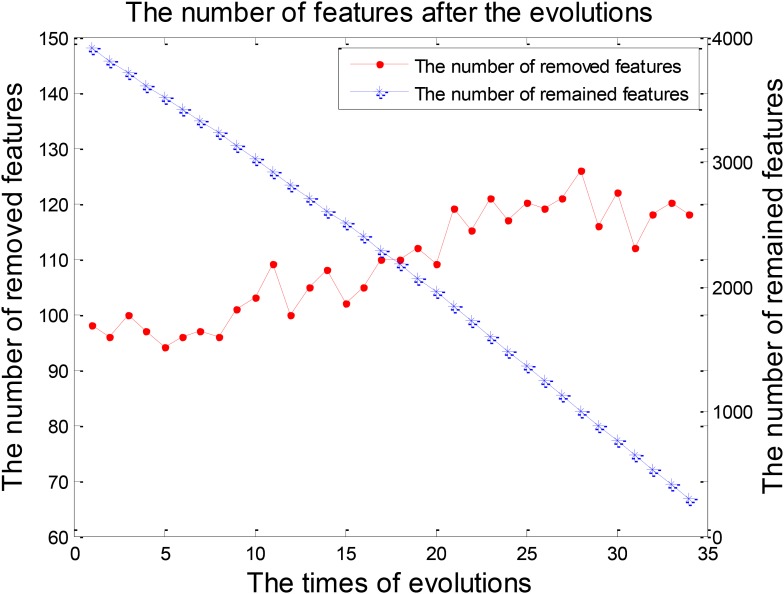
The numbers of features after each evolution in the experiment 2.

The process of feature selection in LMCI/AD classification was plotted in **Figure 7**. The number of removed features showed an overall upward trend, while the number of remained features exhibited a trend of linear decline. There were 293 features left after finishing the 34th evolution, which formed the optimal feature set for the further analysis of progression from LMCI to AD.

**FIGURE 8:**
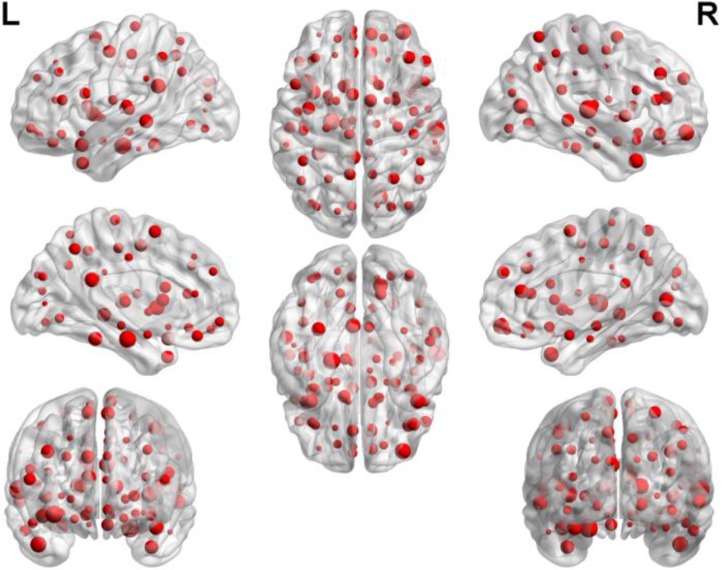
The locations of brain regions in the experiment 2.

We were able to explore the most discriminative brain regions by counting the high-frequency FCs. The disease-related brain regions in LMCI/AD classification were ranked in the **Table 3** and the ones exceeding the frequency of 10 were listed as follows: superior temporal gyrus (STG.R), parahippocampal gyrus (PHG.L), middle frontal gyrus, orbital part (ORBmid.R), calcarine fissure and surrounding cortex (CAL.R), insula (INS.L), temporal pole: middle temporal gyrus (TPOmid.R), and posterior cingulate gyrus (PCG.L). Similarly, some subregions of the temporal lobe and insula showed higher frequencies than other brain regions, suggesting the temporal lobe and insula made greater contributions to the evolution of AD. **Figure 8** described the locations of brain regions.

**Table 3:** The frequencies of the most discriminative brain regions in the experiment 2.

Frequency	Brain region
14	STG.R
13	PHG.L
12	ORBmid.R
11	CAL.R INS.L TPOmid.R PCG.L
10	ACG.R FFG.L TPOsup.L MTG.L

## Discussion

### Classification Effect

In this paper, we propose an advanced framework of EWRSVMC based on resting-state fMRI data to accurately classify different stages of AD. Resting-state fMRI is an effective tool for exploring the dynamical changes in human brain because of the high temporal and spatial resolutions (Lee M.H. et al., 2016). In addition, to the best of our knowledge, no investigation is available about the EWRSVMC in AD studies using brain imaging data. The EWRSVMC is able to efficiently perform EMCI/LMCI and LMCI/AD classifications with the high accuracies of 90 and 88.89%, sensitivities of 90.9 and 85.71%, specificities of 88.89 and 90.9% respectively. The results of two groups of experiments demonstrate the availability of novel EWRSVMC algorithm for early detection of AD and the potential of resting-state fMRI for identification of the transition from EMCI to LMCI to AD.

The ML techniques have received increasingly growing attentions recently in imaging data (Zeng et al., 2014; Wang et al., 2017), and have been shown to be a reliable method to diagnose different cognitive stages of AD using neuroimaging data. Jiang et al. (2014) achieved a high accuracy around 80% for 56 EMCI versus 44 LMCI combining a sparse learning with the SVM classifier. Prasad et al. (2015) reported the accuracy of 63.4% for 74 EMCI vs. 38 LMCI using the SVM classifier with the feature set of the fiber network measures (FIN) and the flow network measures (FLN). Mahjoub et al. (2018) combined the proposed deep similarity network architectures with the single SVM classifier utilizing the cross-validation method to classify 41 AD from 36 LMCI with a classification accuracy peaking at 77.92%.

The majority of ML methods had the slightly lower classification performances especially classifying EMCI from LMCI because of image noise and small-sample size of data. In addition, a great deal of studies have paid more attention to the classification but rarely explored disease-related brain regions underlying the AD evolution. To address these issues, a new framework of EWRSVMC using the FCs as the raw features was presented in this paper. The output of EWRSVMC is a weighted average of the outputs of SVMs, which could further reduce classification error rate compared to some previous methodologies. Additionally, Due to the high dimensionality of feature space, the complexity of the algorithm is likely to be increased and the performance of model estimation is degraded. Accordingly, a method of evolution is employed to dynamically eliminate the redundant features and the features in the optimal EWRSVMC are regarded as the optimal features. Moreover, disease-related brain regions could be found out by identifying these features with high discriminative ability, which provides new insights in the pathology of AD.

The issue of overfitting is a major concern in the training process of our EWRSMC algorithm and more details about it are discussed here. In order to building an individual SVM classifier in EWRSVMC, the training set was randomly chosen out from the all experimental dataset and 62 FCs was randomly chosen out from total 4005 FCs as input features. Because of the randomness of samples and features, each SVM base classifier is greatly different from others, which could reduce the effects of overfitting. Furthermore, the EWRSVMC shows a good classification performance in the test set, suggesting a low risk of overfitting phenomenon.

In our proposed EWRSVMC, two hyperparameters, namely the penalty parameter *C* and the kernel bandwidth σ, need to be determined. Initially, we set parameter *C* and σ to *Inf* and 3 to train the individual RBF-SVM classifier. For comparison, we tested different values for *C* and σ and found no considerable changes in terms of the classification performances of the EWRSVMC, suggesting that the proposed EWRSVMC is considerably robust and universal.

### Analysis of Higher-Frequency Brain Regions

In this part, we mainly discussed about four abnormal brain regions, i.e., temporal lobe, insula, superior frontal gyrus, and parahippocampal gyrus respectively.

#### The Temporal Lobe

Some subregions of the temporal lobe had relatively greater frequencies in both EMCI/LMCI and LMCI/AD classifications, indicating that the temporal lobe is likely to play a crucial role in AD progression. The temporal lobe is situated beneath the lateral sulcus on both hemispheres of the human cerebrum (Kiernan, 2012), which is known to be associated with visual memory, language comprehension, emotion association and executive function (Riley et al., 2010; Bell et al., 2011).

Several previous studies have reported the abnormal temporal lobe in AD progression. Younes et al. (2014) found that the volume of medial temporal lobe structures were relevant to time of progress from MCI to AD. Davatzikos et al. (2011) observed the positive baseline Spatial Pattern of Abnormalities for Recognition of Early AD in temporal lobe in patients with MCI who progressed to AD dementia. Stein et al. (2010) observed the temporal lobe volume differences in brain MRI scans of AD patients, MCI patients and healthy elderly participants. Douaud et al. (2013) found that the cerebral atrophy in medial temporal lobe was vulnerable to the AD progression. Blasko et al. (2008) reported the changes of medial temporal lobe atrophy (MTA) through the evolution from cognitive health to MCI and to AD in a prospective cohort of subjects aged 75 years. The discovery of abnormal temporal lobe may help to improve the understanding of AD progression.

#### The Insula

The insula had a relatively higher frequency than other brain regions in both EMCI/LMCI and LMCI/AD classifications as well, indicating that the insula may make a great contribution in the progression of AD. The insula is a crucial hub of the human brain networks and is folded deep in the floor of lateral sulcus (Cauda et al., 2011). It is reported that the human insula is involved in perception, motor control, general cognition and self-awareness (Kang et al., 2011; Chang et al., 2013).

The insula abnormality was reported in numerous previous literatures in AD pathology. Xie et al. (2012) found out the altered functional integration of the insula networks in AD development. Zhu et al. (2014) observed the significantly greater gray matter volume loss in the bilateral insula in the progression of conversion from HC to MCI to AD with a linear trend. Sojkova et al. (2008) reported the longitudinal alterations in regional cerebral blood flow which involved insula and superior temporal regions in AD progression. Hafkemeijer et al. (2012) mentioned that the patients diagnosed with AD exhibited extensive decreases in gray matter volume in insula and temporal lobe. Patel et al. (2013) reported that the default mode network (DMN) regions, e.g., insula and superior temporal gyrus, were significantly affected by AD pathology. The discovery of the insula abnormality may help to illuminate the underlying neuromechanism of AD disorder.

#### The Superior Frontal Gyrus

The superior frontal gyrus possessed a relatively higher frequency compared to other brain regions in the EMCI/LMCI classification, suggesting that the superior frontal gyrus made an important contribution to the evolution from EMCI to LMCI. The superior frontal gyrus (SFG) is situated at the frontal lobe’ superior part and makes up about one third of the prefrontal cortex of the human brain (Li et al., 2013). It has been reported that the superior frontal gyrus is associated with motor functions and cognitive control especially execution within working memory (Chiao et al., 2009; Van den Stock et al., 2011).

We have reviewed a great deal of previous literature about EMCI and LMCI, and found that there were relatively few studies to make inferences about the brain dynamic differences in the cognitive process from EMCI to LMCI. Accordingly, the discovery of abnormal superior frontal gyrus could be clinically helpful for early detection of AD evolution at MCI stage. Lee E.S. et al. (2016) showed the decreased FC in the right superior frontal gurus in patients with LMCI compared with EMCI, which was agreement with our finding.

#### The Parahippocampal Gyrus

The parahippocampal gyrus obtained a higher frequency in 90 brain regions in the LMCI/AD classification, indicating that the parahippocampal gyrus acted a crucial part in the evolution from LMCI to AD. The parahippocampal gyrus is a part of the limbic system (Enatsu et al., 2015; Arnone et al., 2016), which is involved in the memory encoding and retrieval (Puri et al., 2012; Monti et al., 2018).

Several previous studies have reported the parahippocampal gyrus abnormality in AD pathology. Liang et al. (2014) found out the altered amplitude of low-frequency fluctuations in right parahippocampal gyrus from LMCI and AD. Xiang et al. (2013) reported that AD patients showed less activity than MCI patients in the right parahippocampal gyrus during a visual memory task. Yetkin et al. (2006) mentioned that the AD group had less activation in bilateral parahippocampal gyri than the MCI group in a memory-encoding task. Echávarri et al. (2011) found out the significant differences of volumes of the parahippocampal gyrus between the groups with the following order: AD < aMCI < healthy. The discovery of parahippocampal gyrus abnormality may provide assistant for clinical diagnosis of early AD.

### Limitations

The current study is limited by the following two factors. Firstly, we utilized one modality, i.e., RS-fMRI for multiple binary classifications. Nevertheless, there exist other modalities [e.g., cerebrospinal fluid (CSF) and positron emission tomography (PET)] which may also contain commentary information for better classification performance. Secondly, it is crucial to visualize the learned decision process for better understanding the classification approach and gaining clinicalinsights. However, as with most previous AD classification algorithms, the visualization of the learned decision process in our proposed EWRSVMC is not informative, which is still a limitation which is expected to be addressed in the future.

## Ethics Statement

This study was carried out in accordance with the recommendations of National Institute of Aging-Alzheimer’s Association (NIA-AA) workgroup guidelines, Institutional Review Board (IRB). The study was approved by IRB of each participating site, including the Banner Alzheimer’s Institute, and was conducted in accordance with Federal Regulations, the Internal Conference on Harmonization (ICH), and Good Clinical Practices (GCP).

## Author Contributions

X-aB proposed the design of the work and revised it critically for important intellectual content. QX and QS carried out the experiment for the work and drafted part of the work. XL and ZW collected, interpreted the data, and drafted part of the work. All the authors approved the final version to be published and agreed to be accountable for all aspects of the work in ensuring that questions related to the accuracy or integrity of any part of the work are appropriately investigated and resolved.

## Conflict of Interest Statement

The authors declare that the research was conducted in the absence of any commercial or financial relationships that could be construed as a potential conflict of interest.

## Footnotes

1 http://adni.loni.usc.edu/
